# Computed tomography-based imaging biomarker identifies coal workers’ pneumoconiosis

**DOI:** 10.3389/fphys.2023.1288246

**Published:** 2023-11-22

**Authors:** Jaehun Pyo, Ngan-Khanh Chau, Eun-Kee Park, Sanghun Choi

**Affiliations:** ^1^ Department of Bio-Industrial Machinery Engineering, Kyungpook National University, Daegu, Republic of Korea; ^2^ School of Mechanical Engineering, Kyungpook National University, Daegu, Republic of Korea; ^3^ An Giang University, Vietnam National University, Ho Chi Minh City, Vietnam; ^4^ Department of Medical Humanities and Social Medicine, College of Medicine, Kosin University, Busan, Republic of Korea

**Keywords:** severity of pneumoconiosis, ILO classification, wall thickening, increased abnormal regions, reduced blood vessel volume

## Abstract

**Rationale:** The increase in the incidence and the diagnostic limitations of pneumoconiosis have emerged as a public health concern. This study aimed to conduct a computed tomography (CT)- based quantitative analysis to understand differences in imaging results of pneumoconiosis according to disease severity.

**Methods:** According to the International Labor Organization (ILO) guidelines, coal workers’ pneumoconiosis (CWP) are classified into five categories. CT images were obtained only at full inspiration and were quantitatively evaluated for airway structural variables such as bifurcation angle (θ), hydraulic diameter (D_h_), wall thickness (WT), and circularity (Cr). Parenchymal functional variables include abnormal regions (emphysema, ground–glass opacities, consolidation, semi consolidation, and fibrosis) and blood vessel volume. Through the propensity score matching method, the confounding effects were decreased.

**Results:** Category 4 demonstrated a reduced θ in TriLUL, a thicker airway wall in both the Trachea and Bronint compared to Category 0, and a decreased Cr in Bronint. Category 4 presented with higher abnormal regions except for ground–glass opacity and a narrower pulmonary blood vessel volume. A negative correlation was found between abnormal areas with lower Hounsfield units (HU) than the normal lung and the ratio of forced expiratory volume in 1 s/forced vital capacity, with narrowed pulmonary blood vessel volume which is positively correlated with abnormal areas with upper HU than the normal lung.

**Conclusion:** This study provided valuable insight into pneumoconiosis progression through a comparison of quantitative CT images based on severity. Furthermore, as there has been paucity of studies on the pulmonary blood vessel volume of the CWP, in this study, a correlation between reduced pulmonary blood vessel volume and regions with low HU values holds significant importance.

## 1 Introduction

Pneumoconiosis is an occupational disease caused by long-term dust inhalation, leading to chronic and diffuse aseptic lung tissue inflammation, concurrent with pulmonary fibrosis progression (Centers for Disease Control and Prevention, 2012; Artemoval et al., 2016; Perret et al., 2017). Pneumoconiosis can potentially progress into a life-threatening disease; however, an established treatment remains lacking. Specifically, the decline in lung function can cause dyspnea, decreasing the quality of life and can be a potential threat to survival (Wang et al., 1999; Li et al., 2020). Globally, the incidence rate of pneumoconiosis is surging. According to data from 2016, the number of deaths secondary to pneumoconiosis from occupational exposure was approximately 21,500, and the disability-adjusted life years (DALYs) were estimated to be 580,000 years (Collaborators and collaborators, 2020). Additionally, in 2017, the pneumoconiosis mortality rate per 100,000 people was 0.28, and the rate of DALYs per 100,000 people was reported to be 6.64 (Collaborators, 2020).

Although pneumoconiosis can be fatal, well-accepted treatment options remain limited. Thus far, the radiological assessment of pneumoconiosis has largely depended on an objective evaluation of high-quality X-rays (Zosky et al., 2016). However, majority of the conventional methods are based on the subjective assessment of radiologists, that is, failure of accurately identifying small opacities or oversight on assessing the degree of disease progression (J-P et al., 2023). Recently, several studies have demonstrated that CT provides better sensitive results, compared with chest X-rays, implying that CT is considered as an effective method for diagnosing pneumoconiosis (Takahashi et al., 2018; Hayashi et al., 2022). In addition, quantitative computed tomography (QCT)-based analysis is gaining widespread attention as an effective tool that can more accurately and objectively assess alterations secondary to pneumoconiosis (Liu et al., 2002; Xia et al., 2012). The QCT-based image analysis allows for estimation of the precise location and lesion size and structural lung changes, playing an essential role in the accurate evaluation and monitoring of pneumoconiosis.

The QCT approach has been used as an objective tool for radiological analysis of different lung diseases. This is attributed to the recent advancements in CT image processing technology that allows for regional quantitative metrics extraction, being utilized for pulmonary disease-related research on conditions such as chronic obstructive pulmonary disease (COPD), asthma, and diseases related to particulates such as cement dust (Choi et al., 2014; Choi et al., 2015; Choi et al., 2017; Kim et al., 2020). The QCT measurements allow for the visualization of intricate radiological structures in detail, which further enables disease progression tracking over time (Kim et al., 2022). It could also provide quantitative measurement of the lesion location and size, including the structural and functional changes in the lungs (Park et al., 2021). Therefore, the QCT plays a critical role in the diagnosis, treatment, and research of pneumoconiosis.

This study aimed to assess airway structural and pulmonary functional changes among coal workers’ pneumoconiosis (CWP) according to disease severity via QCT-based imaging metrics. Additionally, through the analysis of pulmonary blood vessel volume in CWP, a topic that has rarely been investigated, we will reveal the relationship between pneumoconiosis and blood vessel volume, which has paucity of data. This will allow us to comprehend the radiological alterations according to pneumoconiosis severity. Using the International Labor Organization (ILO) classification, we first defined pneumoconiosis severity, and confounding factors such as age, height, smoking status, and COPD status were controlled using a propensity score matching (PSM) method. According to each ILO category, the samples after PSM were compared in line with the structural and functional changes (Muszynska-Graca et al., 2016; Fu et al., 2019).

## 2 Methods

### 2.1 Study population

The Institutional Review Board of Kosin University Gospel Hospital approved this study (KUGH-2022-02-009). Data from 380 CWP subjects from the Good Morning Hospital (Miryang, Republic of Korea) were retrospectively collected. CWP was diagnosed through chest X-ray examinations. Spirometric measurements for pulmonary function tests of CWP subjects were conducted following the guidelines of the American Thoracic Society (Miller et al., 2005). Among them, comorbidities such as coalescence of small opacities, bronchitis, inactive tuberculosis, bulla (e), emphysema, bronchiectasis, pleural effusion, cancer (thoracic malignancies excluding mesothelioma), eggshell calcification of hilar or mediastinal lymph nodes, and pleural thickening were manifested by some participants. The CWP subjects take various medications, including the bronchodilators, mucolytics, antihistamines, oxygen therapy, and more.

CWP subjects were classified in accordance with the ILO guidelines, which define lung disease severity. The ILO classification divides opacities into two categories based on their size, that is, small and large opacities (Zosky et al., 2016). For small opacities, the size, density, and distribution of the opacities were classified according to a scale from Category 0 (no symptoms) to Category 3 (severe). Conversely, large opacities are defined as opacities in which the sum of the longest dimensions of opacity exceeds 10 mm. If the opacity size is 50 mm or less, it is classified as Category A. If the size is larger than 50 mm and smaller than the upper right area, it is classified as Category B; however, if it is larger, it is classified as Category C. In this study, all cases of large opacities were considered as Category 4. These categories were used to analyze the relationship between variables in relation to pneumoconiosis.

A Siemens Somatom Scope CT scanner was utilized. The scan was conducted with a detector configuration of 16 × 1.2 mm, a rotation time of 1 s, a pitch of 1.5, a slice thickness of 3 mm, and a peak kilovoltage of 110 kVp. Detailed imaging protocol information can be found in Table 1. In this study, the slice thickness of the images was relatively large, but its size was consistently employed to all participants. For 380 CWP subjects, CT images were acquired at full inspiration and segmented using the software AVIEW (Corline Soft, Co., Ltd., Seoul, Republic of Korea). Then, to attenuate the effects of confounding factors such as age, sex, height, COPD, and smoking status, a PSM method was used. We applied the PSM method to Categories 1, 2, and 4 as the sample size was limited in the Category 3 (N = 9). The details for the PSM method are described in the statistical analysis subsection.

**TABLE 1:** Show the scanner and scanning protocol used for coal workers’ pneumoconiosis; mAs, milliampere-seconds.

Scanners and scanning protocol	
Institution	Good Morning Hospital
Scanner model	Siemens Somatom Scope
Scan type	Spiral
Rotation time (s)	1
Detector configuration	16 × 1.2 mm
Pitch	1.5
Peak kilovoltage (kVp)	110
mAs	90
Dose modulation	Care dose ON
Reconstruction algorithm	B41S
Thickness (mm)	3
Iterative reconstruction	No selection

### 2.2 CT-based airway structural variables

**FIGURE 1:**
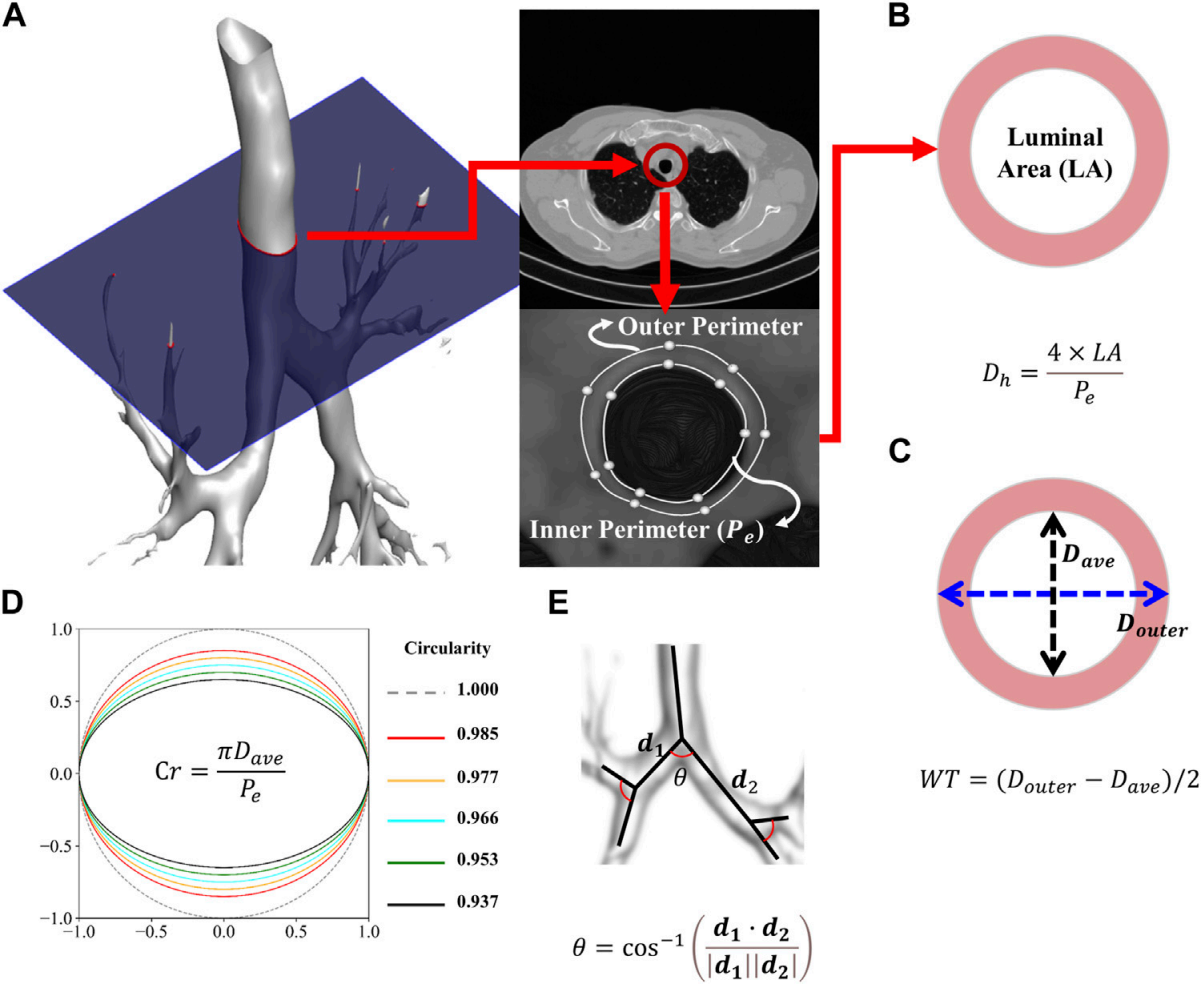
Procedure for extracting the inner and outer perimeters **(A)**. Schematic of the average and hydraulic diameter **(B)**. Schematic of the wall thickness **(C)**. Schematic of the circularity **(D)**. Schematic of the bifurcation angle **(E)**.

Airway structural variables such as one-dimensional (1D) airway skeleton, wall thickness (WT), luminal area, and perimeter of the luminal area ( $P_{e}$ ) were extracted from the CT images using the AVIEW software and in-house post-processing methods (Choi et al., 2015). Furthermore, the bifurcation angle (θ), hydraulic diameter ( $D_{h}$ ), and circularity (Cr) were further calculated using the acquired variables. Using a formula that measures the angle between the vectors of the two daughter branches, the bifurcation angle was calculated via the location information of the branches that can be confirmed through 1D airway skeleton. In Figure 1, the detailed methods for extracting each structural variable and formula are described.

**FIGURE 2:**
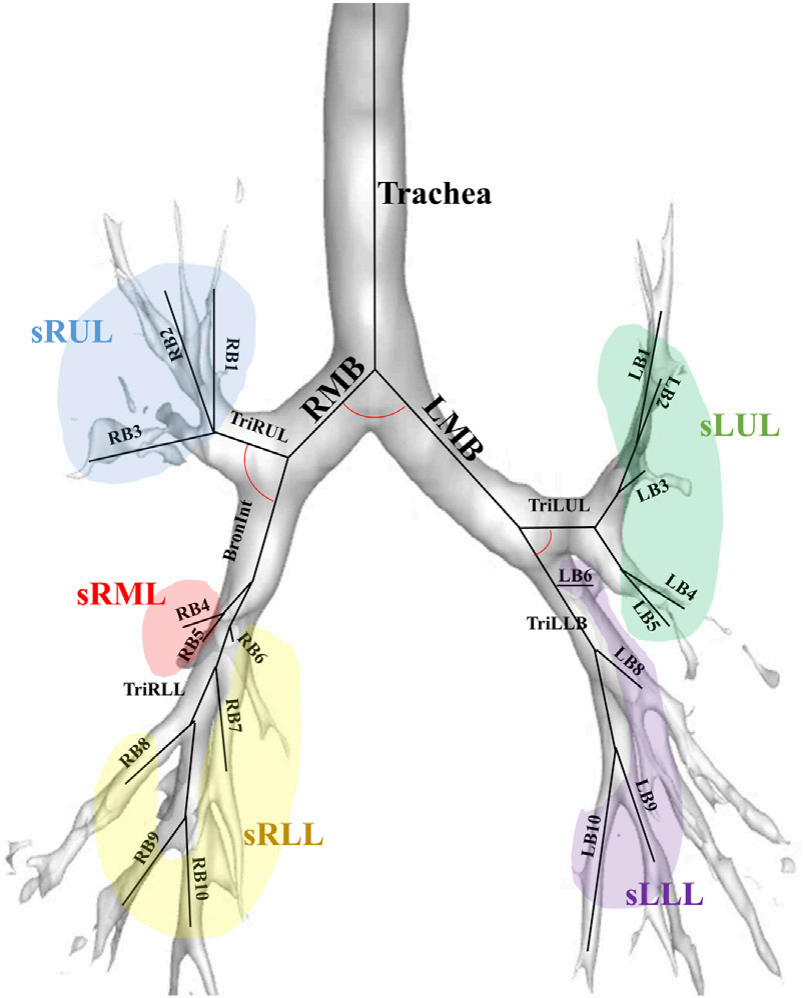
An illustration of the labels of 26 segmental airways including the trachea, left and right main bronchus, Bronint, four trifurcation airways, and five subgroups of lobes; LMB, left main bronchus; RMB, right main bronchus; TriLLB, trifurcation of the left lower lobe; TriLUL, trifurcation of the left upper lobe; TriRLL, trifurcation of the right lower lobe; TriRUL, trifurcation of the right upper lobe; sRUL, subgroup of the right upper lobe; sRML, subgroup of the right middle lobe; sRLL, subgroup of the right lower lobe; sLUL, subgroup of the left upper lobe; sLLL, subgroup of the left lower lobe.

Figure 2 illustrates the labels of 26 segmental airways and five subgroups of lobes to obtain structural variables by branches. From the trachea, left and right main bronchus, Bronint, and four trifurcations of the lobes, values for θ , $D_{h}$ , WT , and Cr were extracted. Additionally, in the five subgroups of the lobes, values for $D_{h}$ , WT , and Cr were obtained .

### 2.3 QCT-based parenchymal functional variables

Functional variables include the ratio of abnormal areas using CT Hounsfield units (HU) and blood vessel volume according to the cross-sectional area ratio of the pulmonary vessels. The abnormal areas through HU include emphysema (under − 950 HU), normal lung ( − 950 to − 701 HU), ground–glass opacity (GGO, − 700 to − 501 HU), semi consolidation ( − 500 to − 201 HU), consolidation ( − 200 to 60 HU), and fibrosis ( − 500 to 0 HU) (Matsuoka et al., 2015; Ho et al., 2021).

To obtain blood vessel volume variables, such as total blood volume (TBV), blood vessel volume of vessels ≤1 mm^2^ in a cross-sectional area (BV1), blood vessel volume of vessels ≤5 mm^2^ in a cross-sectional area (BV5), and blood vessel volume of vessels ≤10 mm^2^ in a cross-sectional area (BV10), the AVIEW software was used (Estepar et al., 2013). The BV1, BV5, and BV10 quantities were then divided using TBV, respectively, to calculate their ratios. The resulting ratios, BV1/TBV, BV5/TBV, and BV10/TBV, were used as indicators to quantitatively represent the degree of pulmonary vessel constriction.

### 2.4 Statistical analysis

We used the PSM method to balance the baseline characteristics between study groups. First, Category 2 (with the lowest number of samples) was set to the treatment group, and Category 0 (with the largest number of samples) to the control group. Between two categories, we performed Greedy one-to-one matching within a caliper of 0.4-fold the standard deviation. Next, Category 0 was set to the treatment group, and Categories 1 and 4 to the control groups. At this time, Greedy one-to-one matching was applied again without the caliper. This could minimize the effect of confounding factors such as age, height, smoking status, and presence of COPD between study groups. Using the standardized mean difference (SMD) of the matched groups, the sample balance was assessed (Zhang et al., 2019). SMD for all pairwise comparisons was calculated using the tableone R package (Yoshida, 2022). We defined it as a case where an absolute value <0.1 is well balanced.

CT scans of CWP subjects often exhibit a remarkable amount of opacities owing to various lesions and disease manifestations (Zhai et al., 2021). These opacities can disrupt the accurate segmentation of small segmental airways within the image. Consequently, during CT image analysis, missing values frequently occur in airway structural variables. Handling of these missing values is crucial as they can greatly impact the accuracy and reliability of data analysis. In this study, to impute these missing values, we utilized the expectation–maximization (E–M) algorithm (Dempster et al., 1977). The E–M algorithm is one of the commonly used methodologies to estimate missing values in the statistical data, effectively ensuring completion of data.

Differences in the structural and functional variables between the category groups were evaluated, matched by each PSM method using the Kruskal–Wallis test (Chan and Walmsley, 1997). A statistically significant difference in the average ranks between multiple groups was confirmed by this test. In cases where statistically significant results were obtained in the Kruskal–Wallis test (*p* ≤ 0.05), a Mann–Whitney U test was performed to specifically confirm which groups had a statistically significant difference. The Mann–Whitney U test compared the average difference between each group and corrected errors that may occur when performing multiple comparisons through the adjustment of the *p*-value using the Bonferroni correction method (Neely et al., 2003; Armstrong, 2014). For a correlation analysis, a heat map was visualized using Pearson’s correlation coefficient for groups that demonstrated significant differences after the Kruskal–Wallis test. The R software (version 4.3.0), Python (version 3.11.3) and SPSS (version 29.0.1.0) were used for all statistical methods.

## 3 Results

### 3.1 Demographic and PFTs’ information

Table 2 and Table 3 illustrate the matched demographic information and pulmonary function information before and after performing PSM, respectively. These tables serve as crucial indicators of how well matching has been conducted between the same entities among CWP subjects of this study. Table 2 provides a demographic information before performing PSM, revealing how differences between entities in the initial dataset appear. Conversely, Table 3 presents information after PSM, demonstrating how confounding factors within the same dataset attained balance. After employing the PSM method, the standardized mean differences for significant confounders such as age, height, smoking status, and COPD presence were found to be 0.050, 0.074, 0.085, and 0.053 respectively. If the absolute standardized mean difference value is <0.1, data are assumed to be well balanced in terms of covariates. The number of female CWP subjects was found to be zero in all groups after the PSM process; hence, they were excluded from the study.

**TABLE 2:** Demographics and pulmonary function test results before propensity score matching. Values are presented as proportion or mean (standard deviation, Std); COPD, chronic obstructive pulmonary disease; SMD, standardized mean difference; FEV_1_, forced expiratory volume in 1 s; FVC, forced vital capacity; BMI, Body Mass Index.

	Category 0	Category 1	Category 2	Category 3	Category 4	SMD
	(N = 110)	(N = 108)	(N = 54)	(N = 10)	(N = 98)	
Female, %	0.0	0.9	0.0	0.0	2.0	0.111
Smoking Participants, %	82.7	81.5	90.7	90.0	91.8	0.171
COPD Participants, %	41.8	60.2	79.6	100.0	85.4	0.747
Age, year	67.1 (7.7)	72.3 (7.5)	73.8 (6.7)	72.2 (8.8)	75.0 (7.2)	0.462
Height, cm	166.4 (6.0)	164.1 (5.8)	164.8 (5.9)	163.4 (5.9)	164.4 (5.9)	0.227
FEV_1_, %predicted	83.5 (16.1)	74.1 (20.0)	63.5 (19.5)	64.8 (21.5)	56.9 (20.9)	0.662
FVC, %predicted	81.2 (13.8)	71.5 (17.6)	65.4 (15.7)	63.8 (10.1)	61.9 (17.9)	0.624
FEV_1_/FVC, %predicted	72.3 (10.2)	69.5 (11.3)	65.7 (14.2)	67.3 (18.4)	60.5 (14.0)	0.411
BMI	24.3 (3.4)	23.7 (3.0)	23.7 (2.8)	24.3 (1.9)	22.7 (2.9)	0.270

**TABLE 3:** Demographics and pulmonary function test results after propensity score matching. Values are presented as proportion or mean (standard deviation, Std); COPD, chronic obstructive pulmonary disease; SMD, standardized mean difference; FEV1, forced expiratory volume in 1 s; FVC, forced vital capacity. BMI, Body Mass Index.

	Category 0	Category 1	Category 2	Category 4	SMD
	(N = 43)	(N = 43)	(N = 43)	(N = 43)	
Female, %	0.0	0.0	0.0	0.0	-
Smoking Participants, %	86.0	88.4	90.7	90.7	0.085
COPD Participants, %	74.4	72.1	76.7	69.6	0.053
Age, year	72.0 (7.5)	72.2 (7.4)	72.7 (6.8)	72.0 (7.3)	0.050
Height, cm	164.5 (6.0)	165.1 (5.9)	165.4 (5.7)	165.2 (5.8)	0.074
FEV_1_, %predicted	78.3 (17.2)	68 (19.1)	62 (18.6)	62.3 (19.6)	0.495
FVC, %predicted	72.8 (12.7)	66.3 (16.1)	66.3 (15.1)	66.8 (18.6)	0.227
FEV_1_/FVC, %predicted	70.7 (11.0)	67.9 (12.4)	64.3 (14.7)	62.6 (13.3)	0.367
BMI	24.5 (3.7)	23.9 (3.1)	23.5 (2.8)	23.0 (2.7)	0.249

### 3.2 Structure variables extracted from segmented airways

**FIGURE 3:**
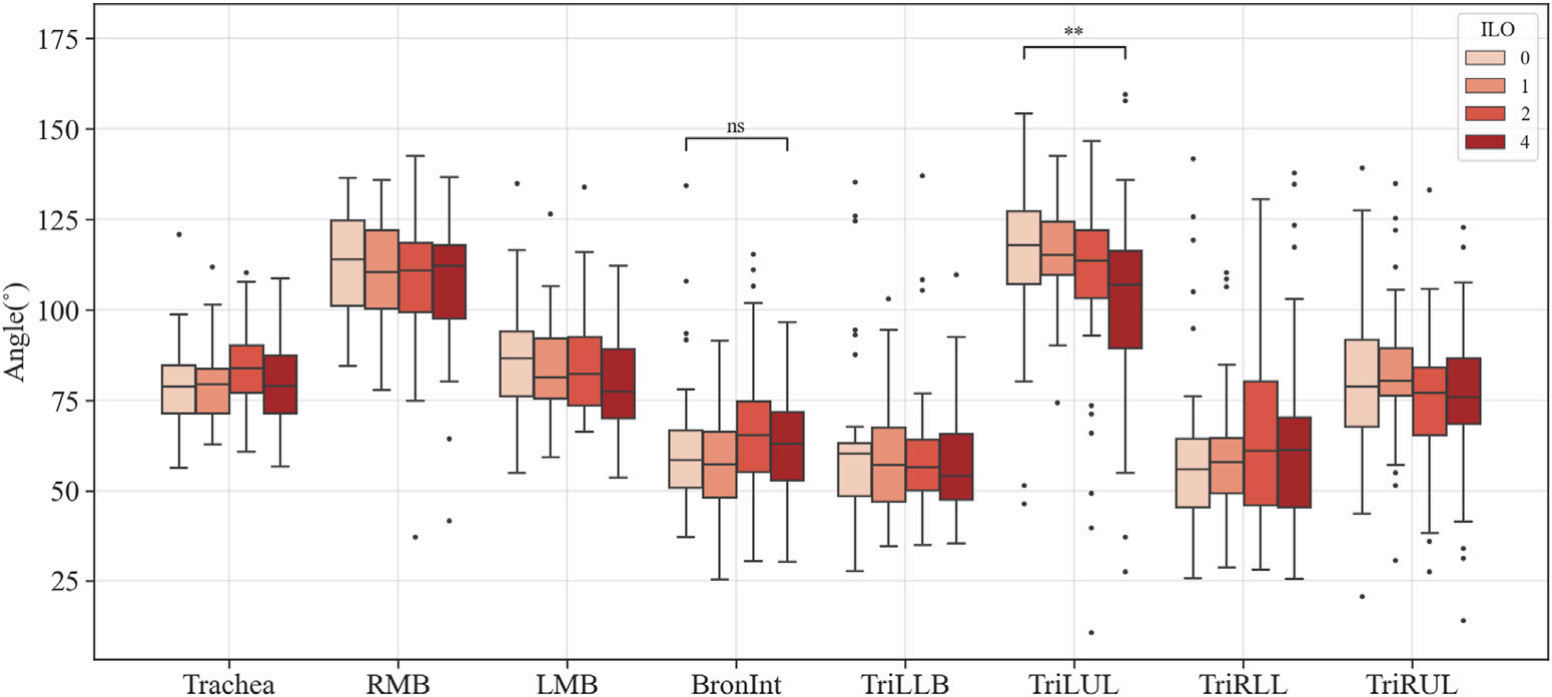
Comparison of the bifurcation angle of coal workers’ pneumoconiosis groups. Values are presented as mean (CI); ns (non-significant, *p* ≥ 0.05); ** (*p* < 0.01). LMB, left main bronchus; RMB, right main bronchus; TriLLB, trifurcation of the left lower lobe; TriLUL, trifurcation of the left upper lobe; TriRLL, trifurcation of the right lower lobe; TriRUL, trifurcation of the right upper lobe.

**FIGURE 4:**
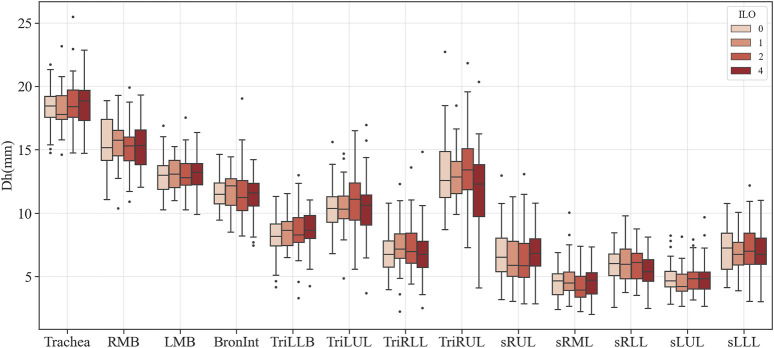
Comparison of the hydraulic diameter of coal workers’ pneumoconiosis groups. Values are presented as mean (CI). LMB, left main bronchus; RMB, right main bronchus; TriLLB, trifurcation of the left lower lobe; TriLUL, trifurcation of the left upper lobe; TriRLL, trifurcation of the right lower lobe; TriRUL, trifurcation of the right upper lobe; sRUL, subgroup of the right upper lobe; sRML, subgroup of the right middle lobe; sRLL, subgroup of the right lower lobe; sLUL, subgroup of the left upper lobe; sLLL, subgroup of the left lower lobe.

**FIGURE 5:**
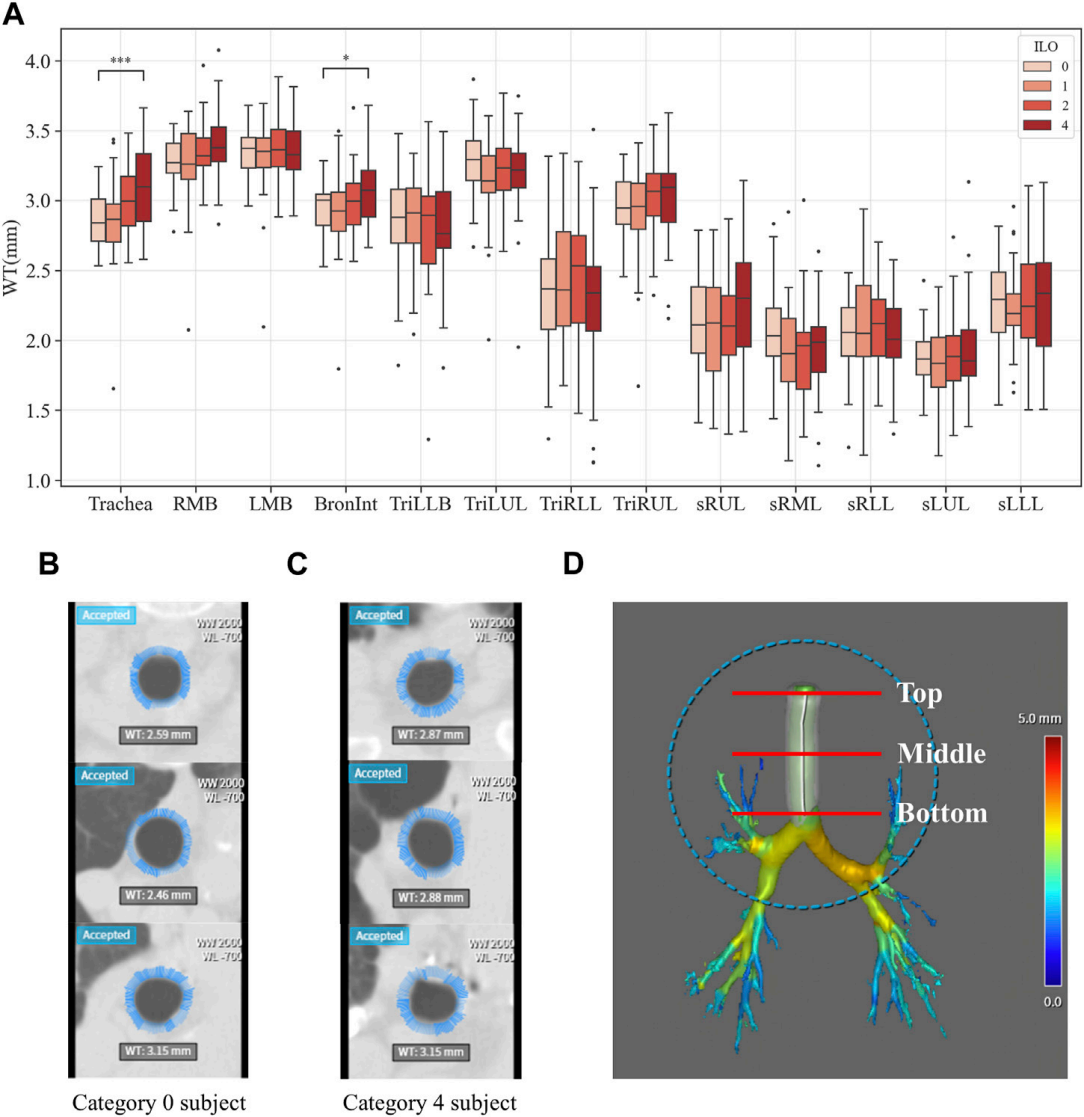
Comparison of wall thickness of coal workers’ pneumoconiosis groups. Values are presented as mean (CI); * (*p* < 0.05); *** (*p* < 0.001). LMB, left main bronchus; RMB, right main bronchus; TriLLB, trifurcation of the left lower lobe; TriLUL, trifurcation of the left upper lobe; TriRLL, trifurcation of the right lower lobe; TriRUL, trifurcation of the right upper lobe; sRUL, subgroup of the right upper lobe; sRML, subgroup of the right middle lobe; sRLL, subgroup of the right lower lobe; sLUL, subgroup of the left upper lobe; sLLL, subgroup of the left lower lobe **(A)**. Comparison of the trachea wall thickness of coal workers’ pneumoconiosis between the two extreme groups (Category 0 vs. 4). Selected participants demonstrate the most similarity in terms of age, height, weight, COPD presence, and smoking status. Their respective attributes are the following: age (75 and 74); height (161 cm and 161 cm); weight (63 kg and 63 kg); COPD (yes for both); and smoking (yes for both). Wall thickness of the Category 0 participant **(B)**; wall thickness of the Category 4 participant **(C)**; section location (including top, middle, and bottom of the trachea) **(D)**.

**FIGURE 6:**
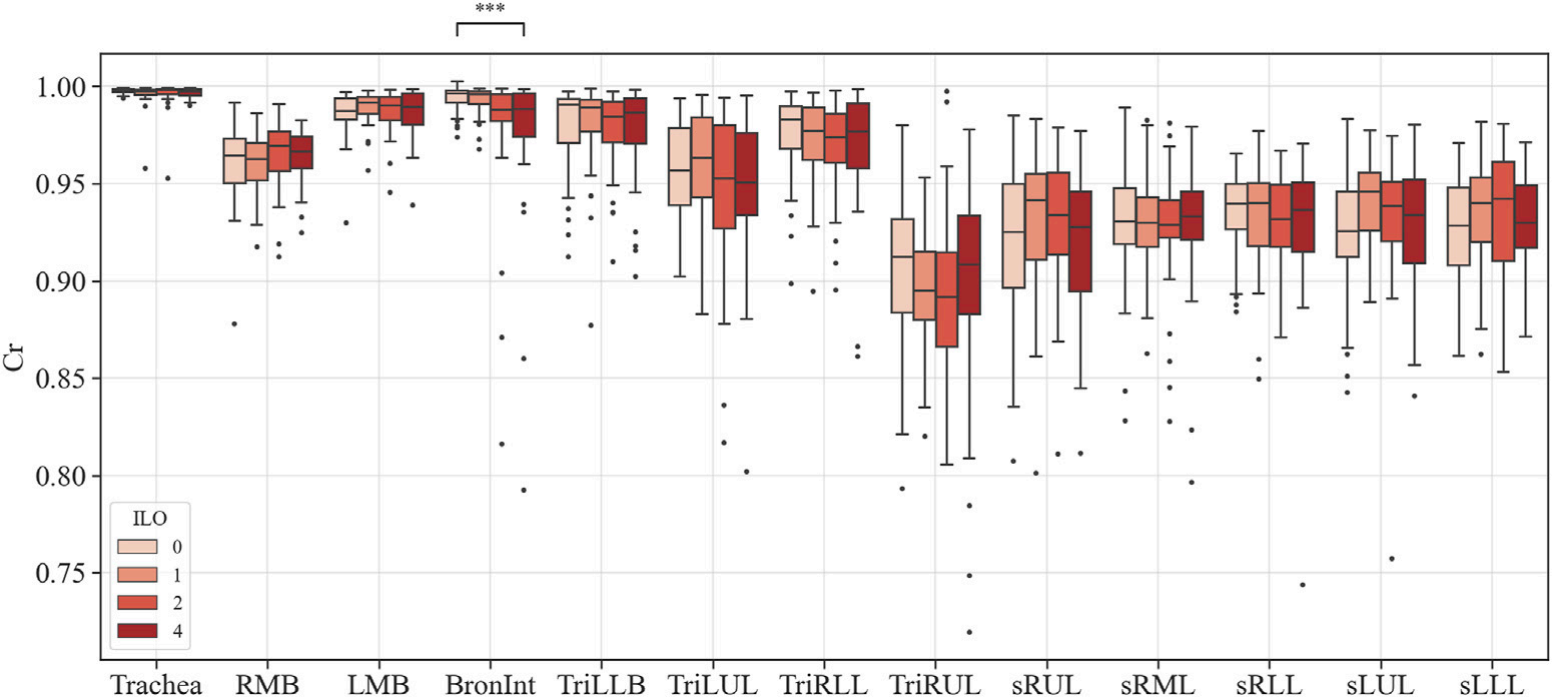
Comparison of circularity of coal workers’ pneumoconiosis groups. Values are presented as mean (CI); *** (*p* < 0.001). LMB, left main bronchus; RMB, right main bronchus; TriLLB, trifurcation of the left lower lobe; TriLUL, trifurcation of the left upper lobe; TriRLL, trifurcation of the right lower lobe; TriRUL, trifurcation of the right upper lobe; sRUL, subgroup of the right upper lobe; sRML, subgroup of the right middle lobe; sRLL, subgroup of the right lower lobe; sLUL, subgroup of the left upper lobe; sLLL, subgroup of the left lower lobe.

Figures 3–6 provide detailed QCT-based analyses for the three primary structural variables such as θ, $D_{h}$ , WT, and Cr. Figure 3 shows the difference in θ among the four categories. Significant differences were noted in Bronint and TriLUL, but only TriLUL exhibited a significant difference between the two extreme groups after the post hoc test. Notably, the Category 4 group in TriLUL had a lower θ than Category 0. Figure 4 illustrates the $D_{h}$ differences among the four categories. No significant difference was observed in any branch in $D_{h}$ . Figure 5A displays the WT differences among categories. In the Trachea and Bronint, group differences were noted. Statistical differences between the extreme groups were apparent in the Trachea and Bronint. In the Trachea, Category 4 exhibited a thicker airway wall than Category 0, and a similar trend was noted in Bronint. Figure 6 highlights the Cr differences across categories. In Bronint, statistical differences were observed, with the Category 4 group showing a lower Cr than Category 0. Figures 5B–D provides a visual difference in WT of the Trachea between Category 0 and Category 4 participants, one of the differences in many structural variables.

### 3.3 Parenchymal function variables of pneumoconiosis participants

**FIGURE 7:**
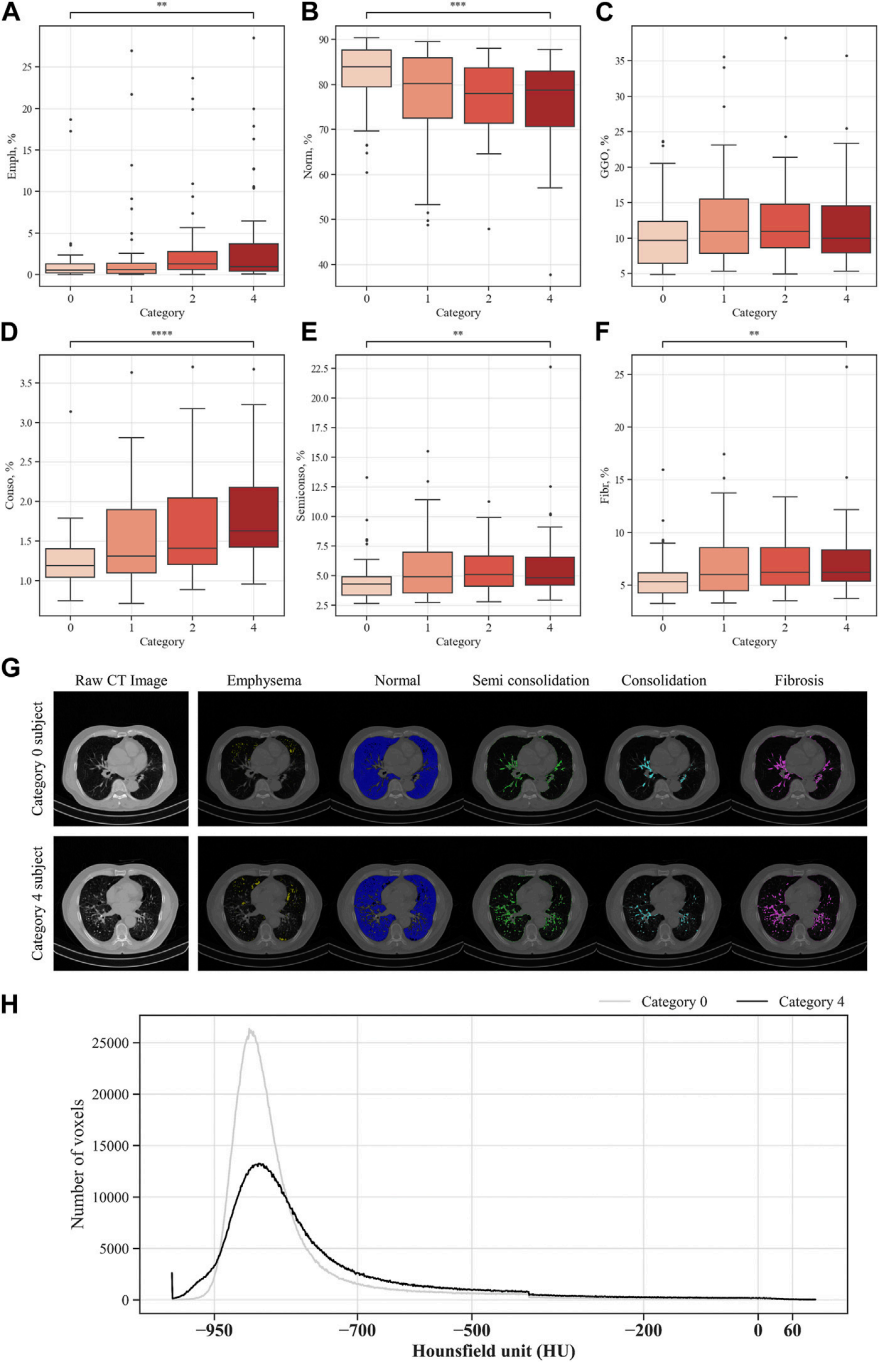
Comparison of parenchymal abnormal regions in coal workers’ pneumoconiosis groups. Values are presented as mean (CI); ** (*p* < 0.01); *** (*p* < 0.001); **** (*p* < 0.0001). Emph, emphysema **(A)**; Norm, normal **(B)**; GGO, ground–glass opacity **(C)**; Conso, consolidation **(D)**; semiconso, semi consolidation **(E)**; Fibr, fibrosis **(F)**. Visual comparison of parenchymal abnormal regions of coal workers’ pneumoconiosis between the two extreme groups (Category 0 vs. 4). Selected participants demonstrate the most similarity in terms of age, height, weight, COPD presence, and smoking status. Their respective attributes are the following: age (75 and 74); height (161 cm and 161 cm); weight (63 kg and 63 kg); COPD (yes for both); and smoking (yes for both). Visualization of abnormal and normal regions for Category 0 participant and Category 4 participant **(G)**. The number of voxels in HU for each category **(H)**.

**FIGURE 8:**
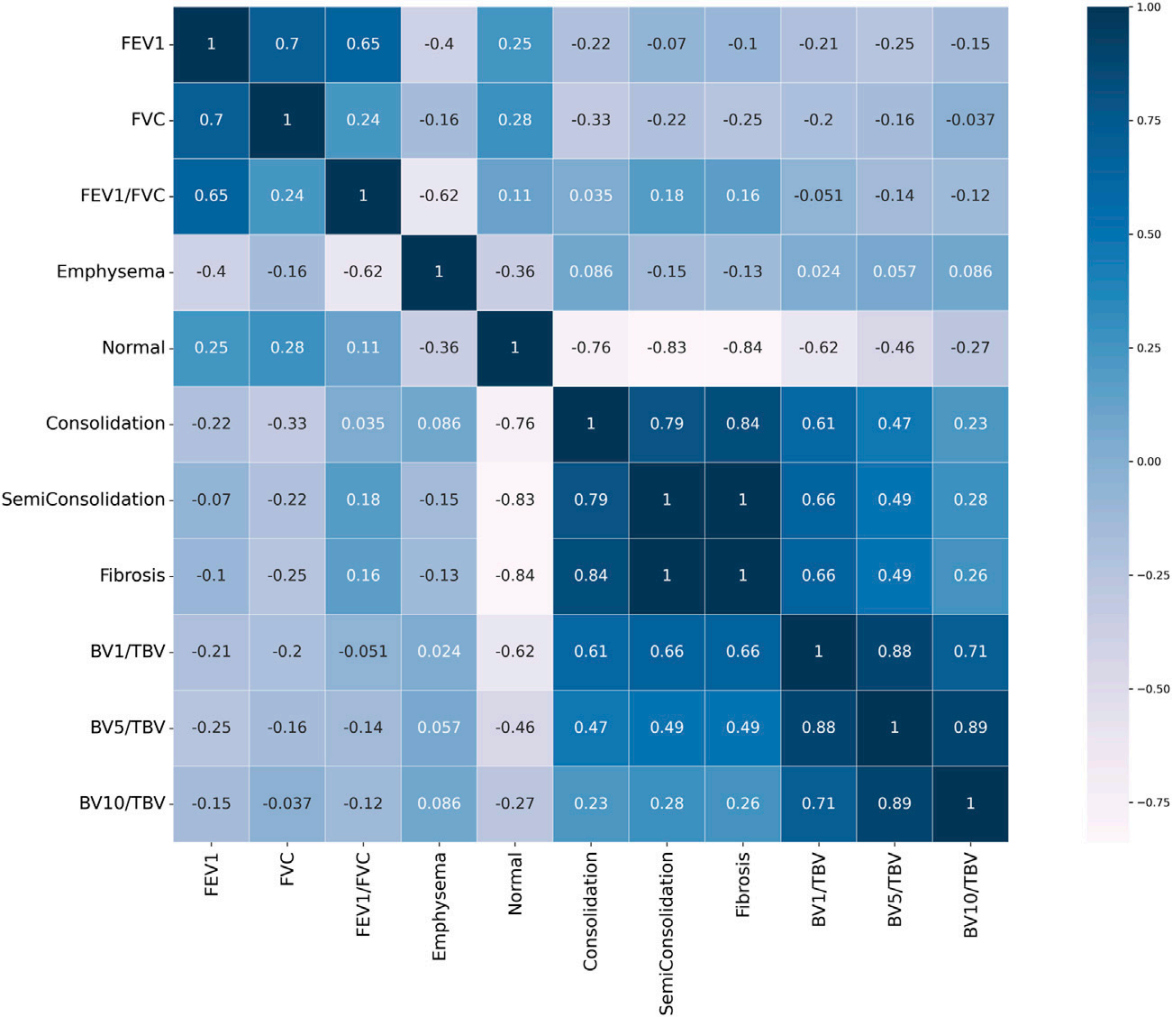
Pearson correlation heat map result. Values close to 1 signify a positive correlation, values close to −1 indicate a negative correlation, and values close to 0 indicate no correlation.

The QCT-based analysis results for abnormal regions in CWP subjects in the four categories are shown in Figures 7A–F. The investigation assessed for several lung conditions including emphysema, normal, GGO, consolidation, semi consolidation, and fibrosis, highlighting differences in severity from Category 0 to Category 4. In this study, Category 4 demonstrated a higher incidence of emphysema compared to those in Category 0. This implies a positive correlation between emphysema severity and the assigned category. For consolidation and semi consolidation, significant differences between categories were also observed, with these conditions being especially more common in Category 4 than those in Category 0. Similarly, fibrosis was more remarkably increased in Category 4 relative to Category 0. As expected, as the severity of the category increases, normal lung portions decreased. Figure 7G shows the visual difference between abnormal and normal regions of a Category 0 participant and a Category 4 participant. Moreover, Figure 7H demonstrates the number of voxels per HU for each category. In Figure 8, a correlation with another variable was noted, and we observed that the emphysema ratio was negatively correlated with the ratio of forced expiratory volume in 1 s (FEV_1_)/forced vital capacity (FVC).

**FIGURE 9:**
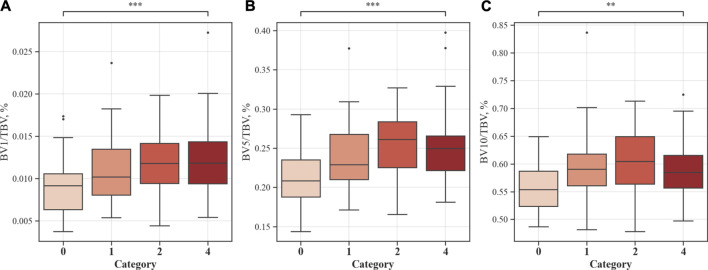
Proportion of blood vessel volumes from the cross-sectional area to total blood vessels. Values are presented as mean (CI); ** (*p* < 0.01); *** (*p* < 0.001). BV1/TBV, proportion of the blood vessel volume of vessels with a cross-sectional area <1 mm^2^ to the total blood vessel volume **(A)**; BV5/TBV, proportion of the blood vessel volume of vessels with a cross-sectional area <5 mm^2^ to the total blood vessel volume **(B)**; BV10/TBV, proportion of the blood vessel volume of vessels with a cross-sectional area <10 mm^2^ to the total blood vessel volume **(C)**.

**FIGURE 10:**
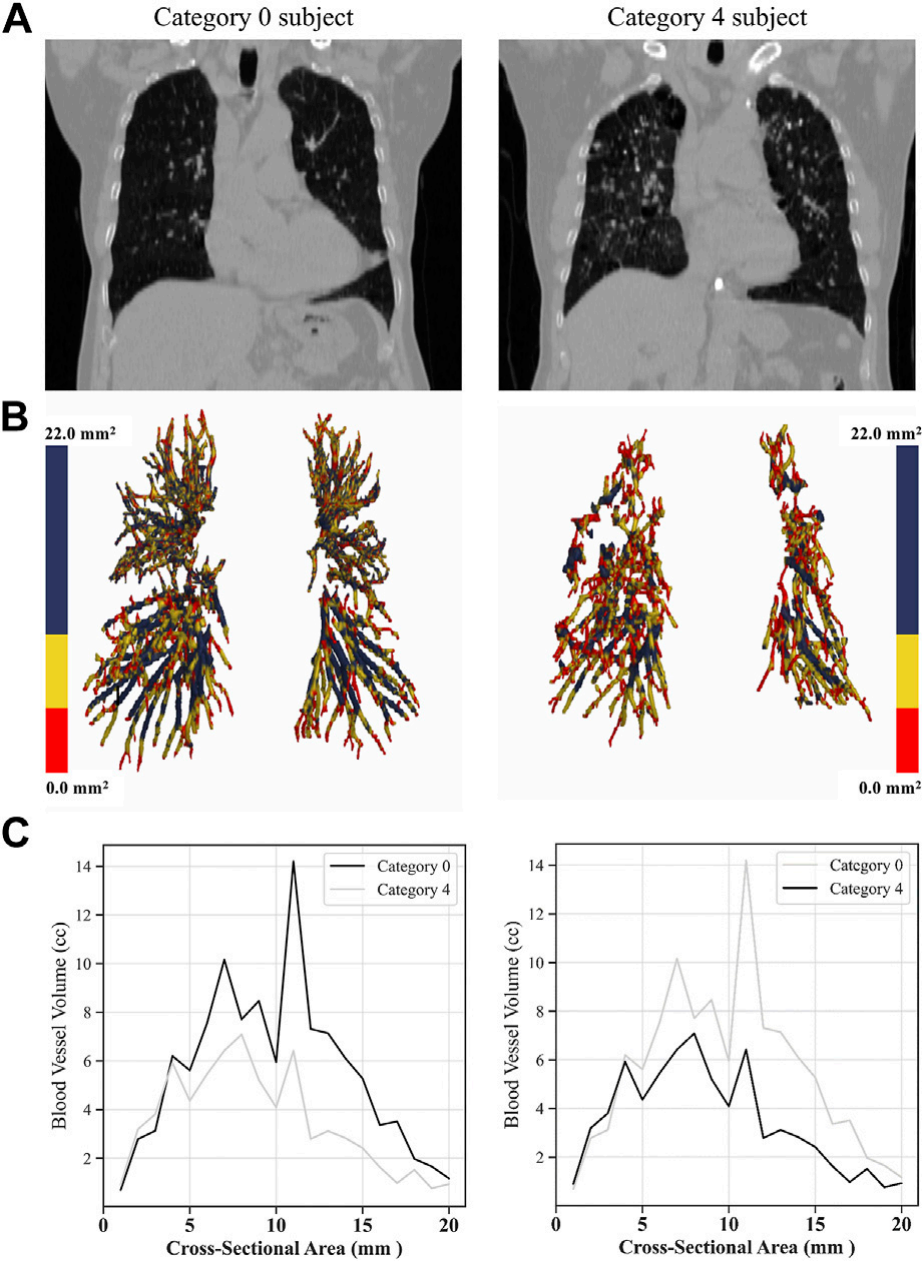
Visual comparison of blood vessel volumes of coal workers’ pneumoconiosis between the two extreme groups (Category 0 vs. 4). Selected participants illustrate the most similarity in terms of age, height, weight, COPD presence, and smoking status. Their respective attributes are the following: age (75 and 74); height (161 cm and 161 cm); weight (63 kg and 63 kg); COPD (yes for both); and smoking (yes for both). Raw CT images of both participants **(A)**; volumetric reconstructions of the pulmonary blood vessels for each participant **(B)**; blood vessel volume in a cross-sectional area from 0 mm^2^ to 20 mm^2^ for each category **(C)**.

Figure 9 shows the blood vessel volume to total blood vessel volume in CWP subjects according to the cross-sectional area ratio. Significant differences were observed between Category 4 and Category 0 in all cross-sectional area ratios BV1/TBV BV5/TBV, and BV10/TBV. As a result, Category 4 revealed a narrower pulmonary vessel cross-sectional area ratio than Category 0. Figure 10B visually shows blood vessels of Category 0 and Category 4 participants. Figure 10C shows the blood vessel volume along the cross-sectional area from 1 mm^2^ to 20 mm^2^. Moreover, a narrower pulmonary blood vessel volume had a positive correlation with consolidation, semi consolidation, and fibrosis and a negative correlation with normal regions (See Figure 8).

## 4 Discussion

In this study, a total of 380 CWP subjects were analyzed. Full inspiratory images of each CWP subjects were collected, and through a quantitative analysis of CT images, structural and functional changes in the lungs were investigated. We aimed to observe the radiological changes in the lungs in accordance with pneumoconiosis progression. To evaluate the severity index, the ILO grade was used. The ILO grade is an internationally recognized measure that defines pneumoconiosis severity (Muszynska-Graca et al., 2016). We analyzed the changes in the structure and functional variables of the lungs according to the ILO group grade. Furthermore, we statistically controlled for confounding factors such as age, height, smoking status, and presence or absence of COPD to create a more objective and reliable comparison between groups. Thus, the PSM method was used to minimize the influence of these confounding factors between comparison groups, while simultaneously enabling a more accurate analysis of radiological characteristics. In addition, to impute missing values in structural variables, E–M algorithm was utilized. Before the application of the E–M algorithm, significant differences were observed in the variables with many missing values based on the Kruskal–Wallis test, especially in the sRML. However, after applying the E–M algorithm, no significant differences were found in these variables (Supplementary Table S5). This confirmed that the application of the E–M algorithm is a more effective method for handling missing values in our research.

Within the analyzed structural variables, multiple regions demonstrated significant variations related to pneumoconiosis severity. The Bronint showed increased values in WT and decreased values in Cr, while TriLUL showed decreased values in θ. The Trachea presented with a higher WT as the severity increases. Given that previous studies have reported increased WT in pneumoconiosis patients who were exposed to agricultural dust, the study findings suggest that excessive dust inhalation, leading to severe pneumoconiosis, may contribute to airway wall thickening in both the trachea and bronchus (Schenker et al., 2009; Marchetti et al., 2014). Besides, the structural variations of WT and Cr in Bronint provides a notion that the Bronint region is a critical area for evaluation of severity progression of pneumoconiosis. In this study, there was no statistically significant difference in the missing values between groups for all airways except the sRUL (Supplementary Table S6). Therefore, we speculated that the relatively coarse CT image resolution may have contributed lack of significant results in the segmented airways, such as the right and left bronchial tubes, were found. Previous imaging-based CT studies showed that most of the airway alterations were observed in the segmental airways rather than large airways such as the trachea and left and right main bronchi (Choi et al., 2015; Choi et al., 2017; Kim et al., 2020). Therefore, to better understand airway structural features in the segmental airways, a prospective future study with standard dose CTs is required.

In the functional variables, emphysema acts as a fundamental factor leading to the most notable functional impairment in CWP subjects (Altinsoy et al., 2016). In our study, a correlation between emphysema and pneumoconiosis severity was found, and particularly, more emphysematous regions were observed in the group under the severe ILO category. Fibrosis refers to areas where normal lung tissue gradually evolves into a fibrous tissue, which causes structural and functional lung damage, and as a result, the lungs gradually lose elasticity and have difficulty performing normal respiratory functions (Thannickal et al., 2004). In our study, a significant amount of fibrosis was noted in the group with severe pneumoconiosis, which supports the pathological characteristics of pneumoconiosis (Centers for Disease Control and Prevention, 2012; Artemoval et al., 2016; Perret et al., 2017). Consolidation and semi consolidation refer to areas with an increased lung tissue density (Liu et al., 2020), which is commonly observed around the blood vessels and airway walls in the group with a severe ILO category. Normal areas were observed the least in the group with a severe ILO category, while it was observed the most in the less severe categories. This suggests a decline in normal areas owing to an increase in the distribution of abnormal areas as pneumoconiosis worsens. GGO is a non-specific term that reflects increased attenuation areas of the lung, including alveolar collapse, interstitial thickening, or air-space disease (Zhang et al., 2020; Cozzi et al., 2021). In our study, no difference between the groups was observed in the Kruskal–Wallis test. Considering the discovery of a GGO pattern in a fibrotic form of pneumoconiosis in previous studies (Chong et al., 2006), it is estimated that there is no GGO distribution with the increase in pneumoconiosis severity. However, to understand this accurately, a longitudinal study is warranted as a follow-up study.

In terms of vessel volume, there is minimal to absent quantitative evaluation of CT-based pulmonary blood vessel volume in CWP subjects. Therefore, in this study, a correlation was performed to identify the complex characteristics of BV in CWP subjects, including a BV analysis according to severity. In Figure 8, emphysema, which is abnormal area with a higher HU than a normal lung, had a negative correlation with FEV_1_/FVC. The pathophysiology of emphysema is characterized by the lung tissue destruction and functional damage, mainly destruction to the bronchial and alveolar walls and capillary layers (Mattison and Christensen, 2006). The FEV_1_/FVC ratio is indicative of overall lung function (Qian et al., 2016). Therefore, in CWP subjects, emphysema development is directly linked to a decline in lung elasticity due to alveolar damage and expansion, thereby leading to a decrease in lung function. In previous studies, observation of emphysema in COPD patients with low FEV_1_/FVC can often be confirmed (Mattison and Christensen, 2006; Turino, 2006; Mouronte-Roibas et al., 2016). Conversely, consolidation, semi consolidation, and fibrosis, which are abnormal areas with a lower HU than a normal lung, had a positive correlation with blood vessel volume. Blood vessel volume is correlated with lung perfusion, a reflection of the capacity for blood flow and oxygen supply in the lungs (Estepar et al., 2013). This is associated with the ability of CWP subjects to absorb oxygen, and a reduction in the vessel volume may signify a lack of oxygen supply. Additionally, increased abnormal regions induce lung tissue damage and inflammation (Thannickal et al., 2004; Liu et al., 2020), which can lead to blood vessel constriction or occlusion, and thus a narrow pulmonary blood vessel volume. This connection emphasizes the complex nature of lung function in pneumoconiosis and highlights the significance of analysis of blood vessel volume and abnormal regions concurrently. However, since researches on blood vessel volume in CWP subjects have been limited or insufficient, abnormal regions and its complex analysis for CWP subjects can be of crucial significance in this study.

In a recent study by Hu X et al., the U-Net neural network was utilized to analyze the texture features of CT images with a slice thickness of 0.75 mm, aiming to identify the stages of pneumoconiosis (Hu X. et al., 2022). Additionally, research by Hu M et al. employed the cascading deep supervision U-Net on CT images with a slice thickness of 5 mm to accurately differentiate patients with pneumoconiosis complicated with pulmonary tuberculosis (Hu M. et al., 2022). While these studies have proven the capability to identify the stages of pneumoconiosis or its coexistence with tuberculosis using CT images, the structural and functional differences according to the severity of pneumoconiosis were not explicitly investigated. In our study, we conducted a quantitative analysis of CT images through an image segmentation, particularly focusing on pulmonary vascular indicators. This approach provided a profound understanding of the intricate characteristics and severity of pneumoconiosis. Such insights are anticipated to offer valuable perspectives in developing diagnostic and therapeutic strategies for pneumoconiosis.

This study yielded various important results; however, there are some limitations. First, because all imaging data of CWP subjects used in the study analysis were images taken in a full inspiratory state, exploration of additional functional variables was limited. For example, in a multi-organ asthma study, the new air-trapping method used full inspiration and full expiration in two CT images (Choi et al., 2014). Considering various breathing states during lung function evaluation can be a vital factor; thus, this limitation should be considered in future research. Second, due to the radiological characteristics of pneumoconiosis, for example, the presence of shadows, a precise analysis of the bronchial ends was difficult. Owing to these limitations, this study did not obtain results for the structural variable differences in the small segment airways. To understand the structural changes of the bronchi in more detail, enhanced imaging processing techniques or other approaches may be warranted. Lastly, since this study utilized a cross-sectional study design, tracking changes over time was impossible. This limited our understanding of the dynamic changes related to pneumoconiosis progression. Also, this study only targeted CWP subjects, and a comparative analysis with a normal group was not conducted. A longitudinal study may allow for observation of lung changes over time and foster understanding of the lung changes of both CWP subjects and a normal group more comprehensively.

## Figures and Tables

**Table:** 

**DALYs**	The disability-adjusted life years
**QCT**	Quantitative computed tomography
**COPD**	Chronic obstructive pulmonary disease
**CWP**	coal workers’ pneumoconiosis
**ILO**	International labor organization
**PSM**	Propensity score matching
**KUGH**	Kosin university gospel hospital
**1D**	One dimensional
**WT**	Wall thickness
$P_{e}$	Perimeter of the luminal area
θ	Bifurcation angle
$D_{h}$	Hydraulic diameter
**Cr**	Circularity
**HU**	Hounsfield units
**GGO**	Ground-glass opacity
**TBV**	Total blood volume
**BV1**	Blood vessel volume of vessels ≤1 mm^2^ in a cross-sectional area
**BV5**	Blood vessel volume of vessels ≤5 mm^2^ in a cross-sectional area
**BV10**	Blood vessel volume of vessels ≤10 mm^2^ in a cross-sectional area
**RMB**	Right main bronchus
**LMB**	Left main bronchus
**RUL**	Right upper lobe
**RML**	Right middle lobe
**RLL**	Right lower lobe
**LUL**	Left upper lobe
**LLL**	Left lower lobe
**Bronint**	Right intermediate bronchus
**TriLLB**	Trifurcation of left lower lobe
**sRUL**	Subgroup of the right upper lobe
**sRML**	subgroup of the right middle lobe
**sRLL**	subgroup of the right lower lobe
**sLUL**	Subgroup of the left upper lobe
**sLLL**	Subgroup of the left lower lobe
**FEV**_**1**_	Forced expiratory volume in 1 s
**FVC**	Forced vital capacity
**mAs**	milliampere-seconds
**SMD**	Standardized mean difference
**SD**	Standard deviation
**CI**	Confidence interval
**BMI**	Body mass index
**Emph**	Emphysema
**Norm**	Normal
**Conso**	Consolidation
**Semiconso**	Semi consolidation
**Fibr**	Fibrosis

## Data Availability

The original contributions presented in the study are included in the article/Supplementary Material, further inquiries can be directed to the corresponding authors.
