# Supplementary material for: Computed tomography-based imaging biomarker identifies coal workers’ pneumoconiosis
Source: Front Physiol. 2023 Nov 22;14:1288246. doi: 10.3389/fphys.2023.1288246 (PMC10702505; doi:10.3389/fphys.2023.1288246)
Supplement: Supplementary file 1 [file Table1.DOCX]

Supplementary Material

Computed Tomography-Based Imaging Biomarker Identifies Coal Workers’ Pneumoconiosis

Jaehun Pyo, BS^1†^, Ngan-Khanh Chau, MS^2,3^, Eun-Kee Park, PhD^4*,^ Sanghun Choi, PhD^2*^

*** Correspondence:**

Sanghun Choi, PhD, School of Mechanical Engineering, Kyungpook National University, Address: 80 Daehak-ro, Buk-gu, Daegu 41566, Republic of Korea. Tel: (8253) 950-5578, Fax: (8253) 950-6550, Email: s-choi@knu.ac.kr

Eun-Kee Park, PhD, Department of Medical Humanities and Social Medicine, College of Medicine, Kosin University, Address: 262 Gamcheon-ro, Seo-gu, Busan 49267, Republic of Korea, Tel: (8251)-990-5424, Fax: (8251)-241-5458, Email: [eunkee.park@gmail.com](mailto:eunkee.park@gmail.com)

# Supplementary Tables

| Structure variable | Category 0 | Category 1 | Category 2 | Category 4 | P–value |
| --- | --- | --- | --- | --- | --- |
|  | (N = 43) | (N = 43) | (N = 43) | (N = 43) |  |
| Bifurcation angle, ˚ | | | | | |
| Trachea | 79.6 (11.7) | 79.7 (10.6) | 84.4 (10.5) | 79.7 (12.5) | 0.077 |
| RMB | 112.6 (13.7) | 109.9 (14.8) | 107.8 (17.7) | 106.8 (18.5) | 0.521 |
| LMB | 87.3 (15.7) | 84.0 (13.7) | 84.6 (14.6) | 80.3 (14.5) | 0.149 |
| BronInt | 62.1 (18.2) | 56.5 (14.4) | 68.2 (18.3) | 61.8 (14.3) | 0.021 |
| TriLLB | 61.8 (23.1) | 58.9 (15.8) | 60.2 (19.2) | 57.7 (15.7) | 0.854 |
| TriLUL | 114.4 (20.1) | 116.3 (14.2) | 107.9 (27.3) | 101.8 (27.2) | 0.008 |
| TriRLL | 59.4 (25.3) | 59.9 (18.6) | 65.1 (25.4) | 64.5 (26.3) | 0.490 |
| TriRUL | 80.0 (24.3) | 82.5 (19.7) | 75.5 (20.8) | 75.8 (22.5) | 0.315 |
| Luminal hydraulic diameter, mm | | | | | |
| Trachea | 18.3 (1.5) | 18.2 (1.6) | 18.6 (1.9) | 18.8 (1.8) | 0.259 |
| RMB | 15.6 (2.1) | 15.6 (1.7) | 15.1 (1.8) | 15.2 (1.8) | 0.469 |
| LMB | 13.0 (1.5) | 13.1 (1.2) | 13.0 (1.4) | 13.1 (1.4) | 0.850 |
| BronInt | 11.6 (1.2) | 11.7 (1.4) | 11.6 (2.0) | 11.4 (1.5) | 0.681 |
| TriLLB | 8.2 (1.5) | 8.5 (1.2) | 8.6 (1.9) | 8.7 (1.4) | 0.364 |
| TriLUL | 10.5 (1.8) | 10.4 (1.9) | 11.0 (2.1) | 10.5 (2.4) | 0.469 |
| TriRLL | 6.9 (1.6) | 7.4 (1.9) | 7.3 (1.8) | 6.9 (2.2) | 0.328 |
| TriRUL | 13.1 (2.8) | 13.0 (1.9) | 13.3 (2.8) | 12.0 (3.0) | 0.212 |
| sRUL | 6.8 (2.1) | 6.4 (1.8) | 6.4 (2.2) | 6.9 (1.9) | 0.321 |
| sRML | 4.5 (1.1) | 4.7 (1.5) | 4.2 (1.2) | 4.5 (1.3) | 0.298 |
| sRLL | 5.9 (1.3) | 6.2 (1.6) | 6.0 (1.4) | 5.5 (1.4) | 0.319 |
| sLUL | 4.9 (1.1) | 4.5 (1.1) | 4.9 (1.2) | 4.9 (1.3) | 0.418 |
| sLLL | 7.1 (1.8) | 6.9 (1.4) | 7.2 (2.0) | 7.0 (1.9) | 0.916 |

**Supplementary Table 1.** Comparison of QCT-based airway structural variables such as bifurcation angle and luminal hydraulic diameter between classified pneumoconiosis patients after propensity score matching. Values are presented as mean (standard deviation, Std); the Kruskal–Wallis test was performed for a group comparison, and an additional Mann–Whitney–Wilcoxon test two-sided with Bonferroni correction was performed for groups that revealed significant results.

| Structure variable | Category 0 | Category 1 | Category 2 | Category 4 | P–value |
| --- | --- | --- | --- | --- | --- |
|  | (N = 43) | (N = 43) | (N = 43) | (N = 43) |  |
| Wall thickness, mm | | | | | |
| Trachea | 2.9 (0.2) | 2.9 (0.3) | 3.0 (0.3) | 3.1 (0.3) | 0.000 |
| RMB | 3.3 (0.2) | 3.3 (0.3) | 3.3 (0.2) | 3.4 (0.2) | 0.101 |
| LMB | 3.3 (0.2) | 3.3 (0.2) | 3.4 (0.2) | 3.3 (0.2) | 0.880 |
| BronInt | 2.9 (0.2) | 2.9 (0.3) | 3.0 (0.2) | 3.1 (0.3) | 0.037 |
| TriLLB | 2.8 (0.3) | 2.9 (0.3) | 2.8 (0.4) | 2.8 (0.3) | 0.845 |
| TriLUL | 3.3 (0.2) | 3.1 (0.3) | 3.2 (0.3) | 3.2 (0.3) | 0.170 |
| TriRLL | 2.3 (0.4) | 2.4 (0.4) | 2.5 (0.4) | 2.3 (0.5) | 0.398 |
| TriRUL | 3.0 (0.2) | 2.9 (0.3) | 3.0 (0.3) | 3.0 (0.3) | 0.324 |
| sRUL | 2.1 (0.4) | 2.1 (0.4) | 2.1 (0.4) | 2.3 (0.4) | 0.191 |
| sRML | 2.1 (0.3) | 1.9 (0.3) | 1.9 (0.3) | 1.9 (0.3) | 0.155 |
| sRLL | 2.0 (0.3) | 2.1 (0.4) | 2.1 (0.3) | 2.0 (0.3) | 0.413 |
| sLUL | 1.9 (0.2) | 1.8 (0.3) | 1.9 (0.3) | 1.9 (0.3) | 0.776 |
| sLLL | 2.3 (0.3) | 2.2 (0.3) | 2.3 (0.4) | 2.3 (0.4) | 0.868 |
| Circularity | | | | | |
| Trachea | 1.00 (0.00) | 1.00 (0.01) | 1.00 (0.01) | 1.00 (0.00) | 0.257 |
| RMB | 0.96 (0.02) | 0.96 (0.01) | 0.97 (0.02) | 0.96 (0.01) | 0.178 |
| LMB | 0.99 (0.01) | 0.99 (0.01) | 0.99 (0.01) | 0.99 (0.01) | 0.335 |
| BronInt | 0.99 (0.01) | 0.99 (0.01) | 0.98 (0.03) | 0.98 (0.04) | 0.000 |
| TriLLB | 0.98 (0.02) | 0.98 (0.02) | 0.98 (0.02) | 0.98 (0.02) | 0.772 |
| TriLUL | 0.96 (0.02) | 0.96 (0.03) | 0.95 (0.04) | 0.95 (0.04) | 0.450 |
| TriRLL | 0.98 (0.02) | 0.97 (0.02) | 0.97 (0.02) | 0.97 (0.03) | 0.460 |
| TriRUL | 0.91 (0.04) | 0.90 (0.03) | 0.89 (0.04) | 0.90 (0.05) | 0.069 |
| sRUL | 0.92 (0.04) | 0.93 (0.04) | 0.93 (0.03) | 0.92 (0.04) | 0.271 |
| sRML | 0.93 (0.03) | 0.93 (0.03) | 0.93 (0.03) | 0.93 (0.03) | 0.966 |
| sRLL | 0.93 (0.02) | 0.93 (0.03) | 0.93 (0.02) | 0.93 (0.04) | 0.947 |
| sLUL | 0.92 (0.03) | 0.94 (0.02) | 0.93 (0.03) | 0.93 (0.03) | 0.068 |
| sLLL | 0.93 (0.03) | 0.94 (0.03) | 0.94 (0.03) | 0.93 (0.02) | 0.197 |

**Supplementary Table 2.** Comparison of QCT-based airway structural variables such as wall thickness and circularity between classified pneumoconiosis patients after propensity score matching. Values are presented as mean (standard deviation, Std); the Kruskal–Wallis test was performed for a group comparison, and an additional Mann–Whitney–Wilcoxon test two-sided with Bonferroni correction was performed for groups that revealed significant results.

| Function variable | Category 0 | Category 1 | Category 2 | Category 4 | P–value |
| --- | --- | --- | --- | --- | --- |
|  | (N = 43) | (N = 43) | (N = 43) | (N = 43) |  |
| Emph, % | 1.6 (3.8) | 2.6 (5.6) | 3.4 (5.6) | 4.3 (6.5) | 0.008 |
| Fibr, % | 5.7 (2.4) | 6.8 (3.2) | 6.9 (2.5) | 7.4 (3.8) | 0.011 |
| GGO, % | 10.7 (5.2) | 13.0 (7.3) | 12.6 (6.0) | 12.4 (6.5) | 0.240 |
| SemiConso, % | 4.6 (2.1) | 5.6 (2.8) | 5.5 (2.0) | 5.9 (3.4) | 0.033 |
| Conso, % | 1.2 (0.4) | 1.5 (0.6) | 1.6 (0.7) | 1.8 (0.6) | <0.001 |
| Norm, % | 81.7 (7.5) | 77.2 (11.2) | 76.8 (8.2) | 75.4 (10.1) | 0.002 |

**Supplementary Table 3.** Comparison of QCT-based abnormal and normal regions between classified pneumoconiosis patients after propensity score matching. Values are presented as mean (standard deviation, Std); the Kruskal–Wallis test was performed for a group comparison, and an additional Mann–Whitney–Wilcoxon test two-sided with Bonferroni correction was performed for groups that revealed significant results.

| Blood vessel volume proportion | Category 0 | Category 1 | Category 2 | Category 4 | P–value |
| --- | --- | --- | --- | --- | --- |
|  | (N = 43) | (N = 43) | (N = 43) | (N = 43) |  |
| BV1/TBV, % | 0.90 (0.32) | 1.09 (0.40) | 1.21 (0.39) | 1.21 (0.43) | <0.001 |
| BV5/TBV, % | 21.37 (3.77) | 23.73 (4.11) | 25.41 (4.04) | 25.24 (4.72) | <0.001 |
| BV10/TBV, % | 55.81 (4.35) | 59.18 (5.86) | 60.28 (5.87) | 58.96 (5.36) | <0.001 |

**Supplementary Table 4.** Comparison of QCT-based blood vessel volume proportion between classified pneumoconiosis patients after propensity score matching. Values are presented as mean (standard deviation, Std); the Kruskal–Wallis test was performed for a group comparison, and an additional Mann–Whitney–Wilcoxon test two-sided with Bonferroni correction was performed for groups that revealed significant results.

| Bifurcation angle  (θ) | Before | After | Luminal hydraulic diameter (Dh) | Before | After |
| --- | --- | --- | --- | --- | --- |
|  | P–value | P–value |  | P–value | P–value |
| Trachea | 0.077 | 0.077 | Trachea | 0.259 | 0.259 |
| RMB | 0.521 | 0.521 | RMB | 0.469 | 0.469 |
| LMB | 0.149 | 0.149 | LMB | 0.850 | 0.850 |
| Bronint | 0.008 | 0.021 | Bronint | 0.668 | 0.681 |
| TriLLB | 0.328 | 0.854 | TriLLB | 0.393 | 0.364 |
| TriLUL | 0.000 | 0.008 | TriLUL | 0.455 | 0.469 |
| TriRLL | 0.364 | 0.490 | TriRLL | 0.253 | 0.328 |
| TriRUL | 0.017 | 0.315 | TriRUL | 0.146 | 0.212 |
|  |  |  | sRUL | 0.130 | 0.321 |
|  |  |  | sRML | 0.000 | 0.298 |
|  |  |  | sRLL | 0.257 | 0.319 |
|  |  |  | sLUL | 0.268 | 0.418 |
|  |  |  | sLLL | 0.945 | 0.916 |
| Wall thickness  (WT) | Before | After | Circularity  (Cr) | Before | After |
|  | P–value | P–value |  | P–value | P–value |
| Trachea | 0.000 | 0.000 | Trachea | 0.257 | 0.257 |
| RMB | 0.101 | 0.101 | RMB | 0.178 | 0.178 |
| LMB | 0.880 | 0.880 | LMB | 0.335 | 0.335 |
| Bronint | 0.030 | 0.037 | Bronint | 0.000 | 0.000 |
| TriLLB | 0.552 | 0.845 | TriLLB | 0.739 | 0.772 |
| TriLUL | 0.162 | 0.170 | TriLUL | 0.441 | 0.450 |
| TriRLL | 0.348 | 0.398 | TriRLL | 0.419 | 0.460 |
| TriRUL | 0.356 | 0.324 | TriRUL | 0.075 | 0.069 |
| sRUL | 0.054 | 0.191 | sRUL | 0.233 | 0.271 |
| sRML | 0.000 | 0.155 | sRML | 0.017 | 0.966 |
| sRLL | 0.316 | 0.413 | sRLL | 0.964 | 0.947 |
| sLUL | 0.709 | 0.776 | sLUL | 0.057 | 0.068 |
| sLLL | 0.839 | 0.868 | sLLL | 0.159 | 0.197 |

**Supplementary Table 5.** Comparison of p–values before and after performing expectation maximization (E–M) algorithm for missing values. P–value was determined using the Kruskal–Wallis test for group comparison.

| Missing values, % | | Bifurcation angle (θ) | | | | | | | | P–value | | Wall thickness (WT) | | | | | | | | P–value | |
| --- | --- | --- | --- | --- | --- | --- | --- | --- | --- | --- | --- | --- | --- | --- | --- | --- | --- | --- | --- | --- | --- |
|  |  | Category 0 | | Category 1 | | Category 2 | | Category 4 | |  |  | Category 0 | | Category 1 | | Category 2 | | Category 4 | |  |  |
| Trachea | | 0.00 | | 0.00 | | 0.00 | | 0.00 | | - | | 0.00 | | 0.00 | | 0.00 | | 0.00 | | - | |
| RMB | | 0.00 | | 0.00 | | 0.00 | | 0.00 | | - | | 0.00 | | 0.00 | | 0.00 | | 0.00 | | - | |
| LMB | | 0.00 | | 0.00 | | 0.00 | | 0.00 | | - | | 0.00 | | 0.00 | | 0.00 | | 0.00 | | - | |
| Bronint | | 11.63 | | 2.33 | | 13.95 | | 11.63 | | 0.278 | | 4.65 | | 0.00 | | 0.00 | | 0.00 | | 0.108 | |
| TriLLB | | 18.60 | | 23.26 | | 13.95 | | 18.60 | | 0.746 | | 6.98 | | 6.98 | | 4.65 | | 6.98 | | 0.962 | |
| TriLUL | | 32.56 | | 27.91 | | 18.60 | | 25.58 | | 0.521 | | 2.33 | | 0.00 | | 0.00 | | 2.33 | | 0.568 | |
| TriRLL | | 11.63 | | 9.30 | | 6.98 | | 16.28 | | 0.558 | | 4.65 | | 2.33 | | 4.65 | | 2.33 | | 0.875 | |
| TriRUL | | 25.58 | | 27.91 | | 20.93 | | 37.21 | | 0.391 | | 2.33 | | 0.00 | | 0.00 | | 2.33 | | 0.568 | |
| sRUL | |  | |  | |  | |  | |  | | 4.65 | | 2.33 | | 2.33 | | 18.60 | | 0.007 | |
| sRML | |  | |  | |  | |  | |  | | 34.88 | | 51.16 | | 48.84 | | 53.49 | | 0.306 | |
| sRLL | |  | |  | |  | |  | |  | | 2.33 | | 0.00 | | 2.33 | | 2.33 | | 0.797 | |
| sLUL | |  | |  | |  | |  | |  | | 6.98 | | 2.33 | | 4.65 | | 16.28 | | 0.075 | |
| sLLL | |  | |  | |  | |  | |  | | 2.33 | | 6.98 | | 2.33 | | 2.33 | | 0.558 | |
| Missing values, % | Luminal hydraulic diameter (D_h_) | | | | | | | | P–value | | Circularity (Cr) | | | | | | | | P–value | |  |
|  | Category 0 | | Category 1 | | Category 2 | | Category 4 | |  |  | Category 0 | | Category 1 | | Category 2 | | Category 4 | |  |  |  |
| Trachea | 0.00 | | 0.00 | | 0.00 | | 0.00 | | - | | 0.00 | | 0.00 | | 0.00 | | 0.00 | | - | |  |
| RMB | 0.00 | | 0.00 | | 0.00 | | 0.00 | | - | | 0.00 | | 0.00 | | 0.00 | | 0.00 | | - | |  |
| LMB | 0.00 | | 0.00 | | 0.00 | | 0.00 | | - | | 0.00 | | 0.00 | | 0.00 | | 0.00 | | - | |  |
| Bronint | 4.65 | | 0.00 | | 0.00 | | 0.00 | | 0.108 | | 4.65 | | 0.00 | | 0.00 | | 0.00 | | 0.108 | |  |
| TriLLB | 6.98 | | 6.98 | | 4.65 | | 6.98 | | 0.962 | | 6.98 | | 6.98 | | 4.65 | | 6.98 | | 0.962 | |  |
| TriLUL | 2.33 | | 0.00 | | 0.00 | | 2.33 | | 0.568 | | 2.33 | | 0.00 | | 0.00 | | 2.33 | | 0.568 | |  |
| TriRLL | 4.65 | | 2.33 | | 4.65 | | 2.33 | | 0.875 | | 4.65 | | 2.33 | | 4.65 | | 2.33 | | 0.875 | |  |
| TriRUL | 2.33 | | 0.00 | | 0.00 | | 2.33 | | 0.568 | | 2.33 | | 0.00 | | 0.00 | | 2.33 | | 0.568 | |  |
| sRUL | 4.65 | | 2.33 | | 2.33 | | 18.60 | | 0.007 | | 4.65 | | 2.33 | | 2.33 | | 18.60 | | 0.007 | |  |
| sRML | 34.88 | | 51.16 | | 48.84 | | 53.49 | | 0.306 | | 34.88 | | 51.16 | | 48.84 | | 53.49 | | 0.306 | |  |
| sRLL | 2.33 | | 0.00 | | 2.33 | | 2.33 | | 0.797 | | 2.33 | | 0.00 | | 2.33 | | 2.33 | | 0.797 | |  |
| sLUL | 6.98 | | 2.33 | | 4.65 | | 16.28 | | 0.075 | | 6.98 | | 2.33 | | 4.65 | | 16.28 | | 0.075 | |  |
| sLLL | 2.33 | | 6.98 | | 2.33 | | 2.33 | | 0.558 | | 2.33 | | 6.98 | | 2.33 | | 2.33 | | 0.558 | |  |

**Supplementary Table 6.** After applying the propensity score matching method, the proportion of missing values and the chi–square test results. The chi-square test was conducted to evaluate the distribution across all groups. Values are presented as proportion.
